# RNA-seq highlights parallel and contrasting patterns in the evolution of the nuclear genome of fully mycoheterotrophic plants

**DOI:** 10.1186/s12864-018-4968-3

**Published:** 2018-08-09

**Authors:** Mikhail I. Schelkunov, Aleksey A. Penin, Maria D. Logacheva

**Affiliations:** 1 Institute for Information Transmission Problems, Russian Academy of Sciences, Moscow, Russia; 2 A.N Belozersky Institute of Physico-Chemical Biology, Lomonosov Moscow State University, Moscow, Russia; 3 Faculty of Biology, Lomonosov Moscow State University, Moscow, Russia; 4 Skolkovo Institute of Science and Technology, Moscow, Russia; 5 Extreme Biology Laboratory, Institute of Fundamental Medicine and Biology, Kazan Federal University, Kazan, Russia

**Keywords:** Ericaceae, Loss of photosynthesis, Mycoheterotrophic plants, Nuclear genome, Orchidaceae, RNA-seq, Sequencing

## Abstract

**Background:**

While photosynthesis is the most notable trait of plants, several lineages of plants (so-called full heterotrophs) have adapted to obtain organic compounds from other sources. The switch to heterotrophy leads to profound changes at the morphological, physiological and genomic levels.

**Results:**

Here, we characterize the transcriptomes of three species representing two lineages of mycoheterotrophic plants: orchids (*Epipogium aphyllum* and *Epipogium roseum*) and Ericaceae (*Hypopitys monotropa*). Comparative analysis is used to highlight the parallelism between distantly related fully heterotrophic plants.

In both lineages, we observed genome-wide elimination of nuclear genes that encode proteins related to photosynthesis, while systems associated with protein import to plastids as well as plastid transcription and translation remain active. Genes encoding components of plastid ribosomes that have been lost from the plastid genomes have not been transferred to the nuclear genomes; instead, some of the encoded proteins have been substituted by homologs. The nuclear genes of both *Epipogium* species accumulated nucleotide substitutions twice as rapidly as their photosynthetic relatives; in contrast, no increase in the substitution rate was observed in *H. monotropa*.

**Conclusions:**

Full heterotrophy leads to profound changes in nuclear gene content. The observed increase in the rate of nucleotide substitutions is lineage specific, rather than a universal phenomenon among non-photosynthetic plants.

**Electronic supplementary material:**

The online version of this article (10.1186/s12864-018-4968-3) contains supplementary material, which is available to authorized users.

## Background

The capability for photosynthesis is the iconic trait of plants and is of the highest importance to the biosphere. However, some plants, including several thousands of flowering plant species, obtain organic substances from sources other than photosynthesis [1, 2]. These plants acquire organic compounds either from associated fungi (myco-heterotrophy) or by parasitizing other plants. Most of these species combine photosynthesis and heterotrophy, but several hundred species have totally lost photosynthetic ability and become fully heterotrophic. The acquisition of heterotrophic ability has occurred in the evolutionary history of plants more than 50 times [1, 2]. The switch to full heterotrophy leads to profound changes at the phenotypic level (reduction of leaves, loss of green colour, reduction of the vegetation period) that are highly parallel in different lineages. The genotypic alterations that underlie these changes are for the most part unclear. The difficulty of cultivating heterotrophic plants under experimental conditions hampers classic genetic and physiological studies. Advances in DNA sequencing permit the application of a genomic approach to elucidate the genetic changes associated with heterotrophy.

Genetic and genomic studies of heterotrophic plants are currently focused on two aspects. The first is the interaction of parasitic plants with their hosts and their adaptations to parasitism (e.g., [3]. Extensive exchange of transcripts occurs between hosts and parasites [4]. On an evolutionary scale, a large number of horizontal gene transfer (HGT) events from hosts to parasites have been found in the organellar and nuclear genomes of parasitic plants [5, 6]. A recent large-scale survey of HGT in Orobanchaceae showed that the number of these events correlates positively with the degree of heterotrophy [7]. The second aspect is the evolution of organellar (mainly plastid) genomes. As expected, the plastomes of heterotrophic plants are reduced in size due to the loss of genes related to photosynthesis, and may retain only ~ 7.5% of the length of a typical plastome as in the parasitic plant *Pilostyles aethiopica* whose plastid genome is approximately 11 kilobase pairs (kbps) long versus approximately 150 kbps in typical autotrophic plants [8]. Despite the high degree of reduction, the plastomes of non-photosynthetic plants retain genes whose products are involved in translation, specifically transfer RNAs, components of the plastid ribosome and two other genes, *accD* and *clpP* (e.g., [8–12]). Although there are several considerations regarding the retention of the plastome, complete loss of the plastome is also apparently possible. At present, there are two known cases of such loss, one in *Polytomella*, a genus of unicellular algae [13], and one in the parasitic angiosperm *Rafflesia lagascae* [14], although alternative explanations are possible in the latter case, like a highly divergent sequence of the plastid genome or its low copy number per cell [15]. Much less information on the mitochondrial genomes of non-photosynthetic plants is available, although there are indications that these genomes are not as extensively reduced in size [16].

The changes in the nuclear genomes of non-photosynthetic plants have not been well studied. To date, the only work to deeply analyse the nuclear genomes of fully heterotrophic plants was performed in Orobanchaceae, where a fully heterotrophic species, *Orobanche aegyptiaca*, was compared with two of its relatives, one mixotroph with obligatory parasitism and one mixotroph with facultative parasitism [17]. Surprisingly, the authors found evidence for conservation of the pathways responsible for chlorophyll synthesis.

**Fig. 1:**
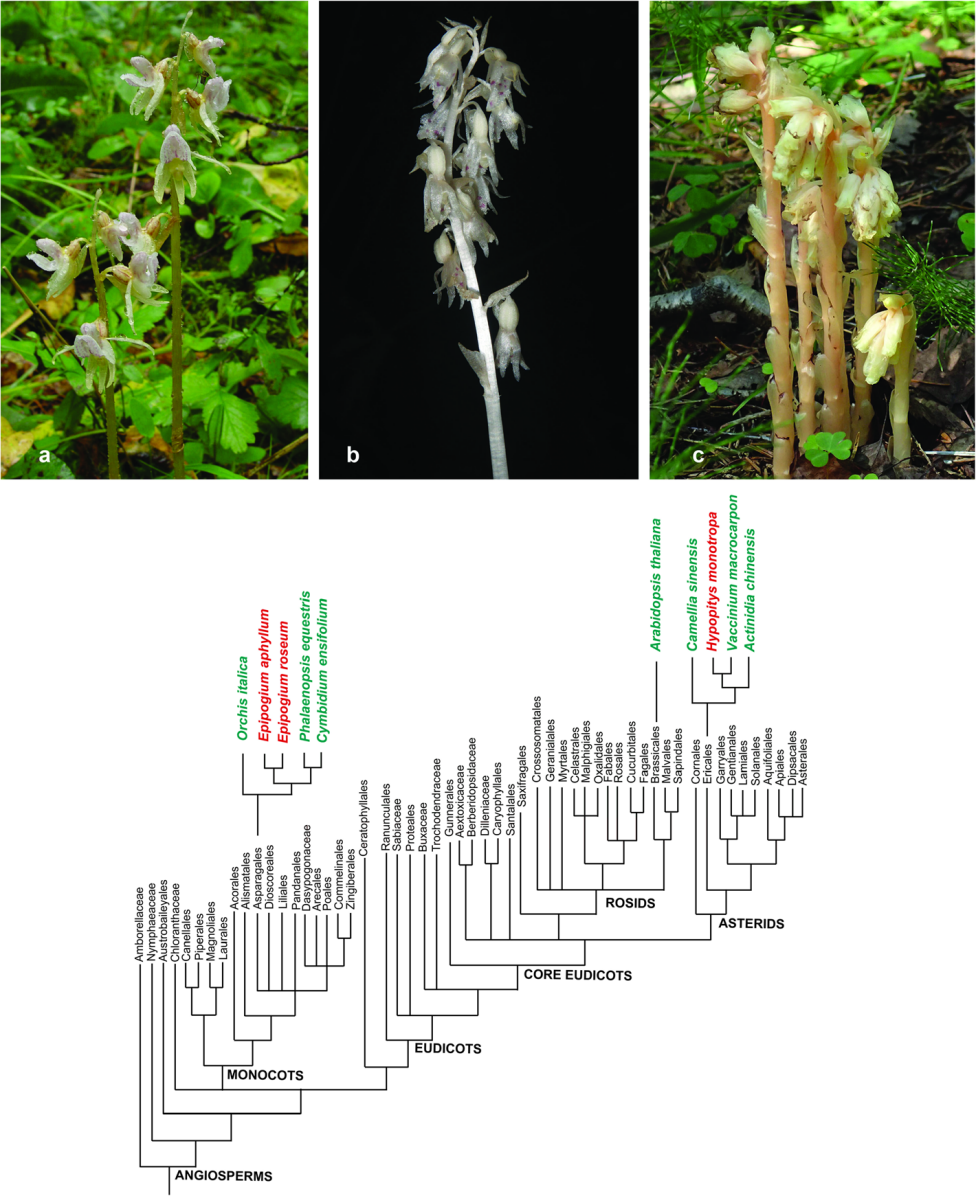
General view and phylogenetic position of the studied species. Upper panel, **a** Epipogium aphyllum, **b** *E. roseum*, **c** *Hypopitys monotropa*. Lower panel: angiosperm phylogenetic tree (redrawn from APG II 2003). The insets demonstrate phylogenetic relationships between heterotrophic species (shown in red) and photosynthetic species (shown in green)

To obtain a more detailed understanding of the evolution of fully heterotrophic plants, in this work, we analyse the nuclear genomes of *Epipogium aphyllum* and *E. roseum* (Orchidaceae) and *Hypopitys monotropa* (Ericaceae) using transcriptome sequencing. These plants, though being very distantly related at phylogenetic level (they belong to the monocots and eudicots, correspondingly), are similar in their appearance (Fig. 1) and ecological characteristics. They are good models for studying the characteristics of fully heterotrophic plants since their plastomes are among the most reduced in size [11, 18] (19–35 Kb versus approximately 150 Kb in typical photosynthetic plants). Therefore, we expect that the nuclear genomes of these species may also differ profoundly from the genomes of photosynthetic plants and will therefore allow us to highlight characteristics that are specific to the genomes of fully heterotrophic species. The nuclear genomes of *E. aphyllum* and *E. roseum* were not studied previously. For *H. monotropa* there is an article, dedicated mostly to the analysis of its plastid genome, which also states that transcripts of some nuclear genes encoding parts of the plastid electron transport chain, photosystems I and II, and ATP-synthase were not found in the transcriptome [19].

## Methods

### Sample collection, library preparation and sequencing

Samples were collected in natural habitats of each species, in the middle of their flowering period. The identification of *E. aphyllum* and *H. monotropa* was carried out by M. D. Logacheva and *E. roseum* – by M.S. Nuraliev. Dried specimen of *E. roseum* is deposited in the herbarium of vascular plants of the Komarov botanical institute (LE) and *H. monotropa* – in the herbarium of Lomonosov Moscow State University (MW). More detailed information on the specimens, including the place and time of collection was reported earlier by Schelkunov et al. [11] for *E. aphyllum* (“White Sea” sample) and *E. roseum* (“Vietnam 2” sample) and by Logacheva et al. [18] for *H. monotropa.* Immediately after collection aerial parts of plants were fixed in RNAlater (Qiagen, Netherlands). Prior to extraction the samples were homogenized in liquid nitrogen. RNA was extracted using Qiagen RNeasy Plant Mini kit (Qiagen, Netherlands) using manufacturer’s instructions. The only modification was the addition of Plant RNA Isolation Aid (ThermoFisher Scientific, USA) to the lysis buffer. To allow the characterization of non-polyadenylated transcripts (e.g., plastid and mitochondrial) we performed depletion of ribosomal RNA using Ribo-Zero plant leaf kit (Illumina, USA). For *H. monotropa* one rRNA depleted and four polyA-RNA libraries were prepared, for *Epipogium* only rRNA depleted libraries, due to the limited availability of the material. All libraries were prepared using RNA Sample prep kit v. 2 (Illumina, USA) following manufacturer’s instructions. The libraries were sequenced on Illumina HiSeq and Illumina MiSeq sequencing machines in paired-end mode. Detailed information on the libraries and sequencing settings, as well as links to on-line databases where the reads are deposited, is provided in Additional file 1: Table S1.

### Choice of datasets of photosynthetic plants

To compare the fully heterotrophic species with typical photosynthetic plants, we obtained data from several RNA-seq experiments and from the genome assemblies of the plants that were available as of 2015. A sequenced transcriptome had to satisfy the following criteria to be used in our study:

1. At least 100 million RNA-seq reads should be sequenced. A study of the transcriptomes of two species of Geraniaceae suggests that the completeness of the assemblies does not increase highly when using more than 100 million of Illumina reads [20].
2. The sequencing should have been performed on Illumina sequencing machines, since they generate reads with the least number of sequencing errors compared to other technologies.
3. The sequenced transcriptome should belong to above-ground parts of a plant, preferably to the inflorescence. This facilitates the comparison with the transcriptomes of *Epipogium* and *Hypopitys*, for which inflorescences were sequenced. Using transcriptomes of some other parts, for example roots, could be inappropriate because they can contain a different set of expressed genes.

In view of these considerations, we chose six photosynthetic species, including three for comparison with *Epipogium* and three for comparison with *H. monotropa*. The names and sources of the datasets on photosynethetic plants are listed in Additional file 1: Tables S1 and S2. In the case of RNA-seq data, we assembled the transcriptomes using exactly the same methods that were used for the transcriptomes of *Epipogium* and *Hypopitys*.

### Transcriptome assembly and postprocessing

Reads were trimmed with Trimmomatic 0.32 [21]. Trimming involved the removal of sequencing adapters, bases with a Phred quality of less than 3 from both the 5′ and 3′ ends of reads, and bases from the 5′ ends of reads starting from a region with 5 consecutive bases with an average score of less than 10 (trimming with a sliding window). Additionally, reads that had average quality of less than 20 after trimming and reads that after trimming became shorter than 30 base pairs (bps) were removed. For species with several sequenced libraries reads were pooled. Assembly was performed using Trinity version r20140717 [22]. We utilized the option of digital normalization provided by Trinity, normalizing the read coverage to 50× – this option highly reduces RAM and CPU usage while almost not affecting the assembly quality [23]. The minimum contig length was set to 100 bps instead of the default value of 200 bps. After assembly, we performed filtration, removing minor isoforms, low-coverage transcripts and contamination. We defined major isoforms as the isoforms to which the highest number of reads (estimated using RSEM [24] in-built in Trinity) were mapped relative to other isoforms. After removing minor isoforms, we filtered low-coverage transcripts by mapping reads to contigs using CLC Assembly Cell 4.2 [25], with a requirement of at least 80% of read’s length mapping with sequence identity at least 98%, and retaining only transcripts with average coverage at least 3 reads per positions. Potential coding sequences (CDSs) in the transcripts were then determined using TransDecoder version r20140704 [23]. The criteria for considering an open reading frame (ORF) to be a potential CDS consisted not only of hexanucleotide frequencies, which are employed in TransDecoder by default, but also all ORFs that possessed domains from the Pfam-A or Pfam-B databases. The minimum CDS length was decreased from the default of 100 amino acids to 30 amino acids. When there were several potential CDSs from a transcript, only the longest one was taken. Then, to remove contamination, we performed BLASTP 2.2.29+ [26] alignment (maximum allowed e-value 10^− 5^, word size 3, the low-complexity filter switched off) of the translated CDS sequences against the NCBI (National Center for Biotechnology Information) NR database and against proteins of related plants whose genomes had been sequenced. A CDS was considered to represent contamination if the BLASTP match with the highest bit score was to a species that was not from Streptophyta (in terms of NCBI Taxonomy). Conversely, if the best match was to Streptophyta member or the sequence presented no BLASTP matches, the CDS was considered to belong to a plant. Statistical parameters of the assemblies, such as N50 values and the number of sequences longer than 1000 bps, were calculated using custom scripts after each filtering step described above. Estimation of the completeness of the assemblies was performed with CEGMA 2.5 [27]. To evaluate gene expression without contamination, we calculated FPKM (Fragments per kilobase per million reads mapped) values using RSEM for transcripts that contained CDSs that were determined not to have arisen from contamination. Minor isoforms of the transcripts were also used for the analysis; the FPKM values provided in Additional file 1: Table S10 are the sums of all gene isoforms.

The assembled transcripts are deposited in the Transcriptome Shotgun Assembly Database (TSA) with accessions provided in Additional file 1: Table S1. Additionally, the longest ORFs from the major isoforms of the transcripts, after the removal of low-coverage transcripts and contamination, are provided in Additional files 2, 3 and 4.

### Transcriptome annotation and gene ontology analysis

Three types of annotations of the CDSs were performed. The first was the computation of 1–1 orthologs from the CDSs of each species by reciprocal alignment with *Arabidopsis thaliana* proteins from TAIR10 (The Arabidopsis Information Resource, 10th release) database [28] using BLASTP (parameters are the same as indicated above). Lists of 1–1 orthologs for *E. aphyllum*, *E. roseum* and *H. monotropa* are provided in Additional file 1: Tables S3, S4 and S5. Also we performed GO annotation using results of BLASTP alignment of all proteins against the NCBI NR (parameters as above) and annotated them with B2G4Pipe 2.5, a command-line wrapper for Blast2GO [29]. Then we assigned KEGG (Kyoto Encyclopedia of Genes and Genomes) Orthology identifiers to the CDSs with the GhostKoala server [30]. The orthogroups in the studied species were calculated separately for Orchidaceae and Ericales using OrthoMCL 2.09 [31] (inflation parameter 1.5).

For GO term enrichment analysis, we used a set of custom scripts written in Perl and R. Utilizing the results of the GO annotation performed with Blast2GO and a graph of GO terms [32], the scripts provide all GO terms corresponding to a gene, including terms that are paternal (i.e., with higher hierarchical levels) to those provided by Blast2GO. Comparison of the numbers of genes with specific GO terms between a pair of species was then performed via a series of Fisher’s exact tests (one for each GO term). GO terms that were not assigned to any genes in either species were excluded from the analysis. After the Fisher’s tests were performed, the scripts performed group comparisons between fully heterotrophic and photosynthetic plants for each GO term, by taking all *p*-values for each pairwise comparison between pairs of species within the group (Ericales or Orchidaceae). The scripts then conducted Bonferroni correction for multiple testing to evaluate the statistical significance of the differences between these groups separately for Orchidaceae and Ericales. Next, another round of correction for multiple hypothesis testing was performed, taking into account the fact that an individual Fisher’s test was performed for each GO term. This correction was performed via the method of Benjamini-Yekutieli [33].

Since our goal was the analysis of nuclear genes, we excluded genes encoded in the plastid and mitochondrial genomes from the enrichment analysis by excluding all genes that were 1–1 orthologs of plastid and mitochondrial genes of *Arabidopsis thaliana.* Some plastid and mitochondrial transcripts may not have been present in the assemblies. To compensate for this, in Additional file 1: Table S10, we indicated a plastid gene as present in the plastome of a studied species not only when it was found through the 1–1 ortholog method, but also when it was present in the annotation of the plastome of that species provided in GenBank (accessions are provided in Additional file 1: Table S2). Because the plastome of *Orchis italica* is not available, we employed the plastome of *Habenaria pantlingiana*, the only species with a characterized plastome from subtribe Orchidiae, which includes *O. italica*.

### Analysis of substitution rates and selective pressure

To estimate the average selective pressure and substitution rates in the genomes of the studied species, we concatenated the sequences of all genes from orthogroups containing exactly one gene from each species. The concatenated sequences were then aligned using TranslatorX 1.1 [34]. As a tool for the alignment of the amino acid sequences by TranslatorX, we used Muscle [35]. The topologies of the phylogenetic trees for Orchidaceae and Ericales were obtained from Peter Stevens’ Angiosperm Phylogeny Website, version 14 [36]. dN/dS and substitution rate estimation was performed based on the alignment and the tree using PAML 4.8 [37], employing a branch model with free ratios, the Gy94_3 × 4 codon model and removal of all columns with at least one gap in the alignment.

To compare the magnitude of the selective pressure acting on individual genes in heterotrophic and autotrophic plants, the sequences of the genes were aligned using TranslatorX and Muscle as described above, and dN/dS ratios were calculated using PAML in the branch model. For the Orchidaceae, two calculations were performed. In the first calculation, one dN/dS was allowed for the branches of autotrophic plants; another dN/dS was allowed for the terminal branches of *E. aphyllum* and *E. roseum*; and a third dN/dS was allowed for the branch of their common ancestor. The second calculation was performed allowing one dN/dS for the common ancestor of *E. aphyllum* and *E. roseum* and a second dN/dS for all other branches, including autotrophic species, *E. aphyllum* and *E. roseum*. The *P*-values for the difference in dN/dS between the terminal branches of *Epipogium* and the branches of autotrophic species were calculated using the likelihood ratio test. Allowing an individual dN/dS for the branch of the common ancestor of *Epipogium* was beneficial, as photosynthetic ability was lost on that branch, and it is unclear whether to group it with autotrophic or heterotrophic species. Similar calculations were performed for Ericales, allowing different dN/dS ratios for the *H. monotropa* branch and other branches the first time and demanding a single dN/dS for all branches the second time. Since *H. monotropa* was the only heterotrophic species from Ericales employed in this study, its terminal branch partially included its autotrophic ancestor. Thus, the values of dN/dS provided for *H. monotropa* in Additional file 1: Table S10 describe selective pressure partially before and partially after the loss of photosynthetic ability. dN/dS was calculated only for genes that were present in all 5 species of Orchidaceae or in all 4 species of Ericales.

### Analysis of protein targeting to organelles

To estimate the number of proteins targeted to various organelles, we predicted transit peptides using TargetP 1.1 [38] and DualPred [39]. Unlike TargetP, DualPred is also capable of predicting proteins that are dually targeted to mitochondria and plastids. TargetP classifies predictions into five “reliability classes”, where the most confident predictions belong to the first class, and the least confident predictions belong to the fifth class. We considered a protein to be potentially targeted to an organelle if its transit peptide exhibited a reliability class of four or less. For DualPred, we considered a protein to be dually targeted to plastids and mitochondria if it presented a dual-targeting score of at least 0.5, as suggested by the first author of DualPred in a personal communication. Prediction of transit peptides was performed only for proteins whose genes exhibited a completely assembled 5′-end according to TransDecoder and were assigned at least one GO term.

To predict the targeting of ribosomal proteins, we utilized a more elaborate technique. It has been demonstrated that some proteins are dually targeted to plastids and mitochondria not because they have one transit peptide that allows them to enter both organelles, but because their mRNAs exhibit alternative translation start sites, resulting in proteins with different transit peptides [40]. To search for transit peptides that may originate from alternative translation, we truncated all of the ribosomal proteins under analysis before the first methionine occurring after the first 25 amino acids (as in [40] and performed TargetP and DualPred analyses for these shortened versions of the proteins as well. Additionally, all alternative isoforms of the ribosomal proteins were analysed. When DualPred suggested that a protein was dually targeted and TargetP suggested either plastid or mitochondrial targeting, we considered this to represent a non-contradictory prediction suggesting dual targeting.

The Pfam families to which the ribosomal proteins belonged were determined by aligning *Arabidopsis thaliana* proteins to all families from the Pfam database, version 31.0 [41], using the HMMER server [42]. The search was performed with the default parameters, and the family that was indicated by the web-service as the “Top Hit” was condidered to be the family this protein belongs to. To identify proteins that belonged to these families in *E. aphyllum*, *E. roseum* and *H. monotropa*, we conducted a search with hidden Markov models of these families using the hmmscan tool from HMMER package, version 3.1b1 [43]. The search was performed among all of the CDSs from transcripts, not only the longest ones, to make detection in polycistronic mitochondrial mRNAs possible. The queries in the search were the hidden Markov model (HMM) profiles of the ribosomal protein families. The subjects were the aforementioned CDSs translated to amino acids. The e-value threshold for a match to be considered significant was set to 10^− 5^. Orthologous proteins in the photosynthetic species employed for comparison were determined as belonging to orthogroups (calculated by OrthoMCL as described above) that contained the proteins identified for *E. aphyllum*, *E. roseum* and *H. monotropa*.

To search for homologs of RECA which may function in plastids of the studied heterotrophic species, we aligned the hidden Markov model of RECA (PF00154 model in the Pfam database) to the CDSs, exactly as with did while searching for the ribosomal proteins. Putative transit peptides were predicted in the same way too.

### Graphic representation of results

Phylogenetic trees were drawn with TreeGraph 2.9.2 [44]. Maps of metabolic and signalling pathways were built on the KEGG site [45] (accessed 22 October 2015).

## Results and discussion

### Characteristics of transcriptome assembly

The statistics of transcriptome assembly are provided in Table 1 (for details, see Additional file 1: Table S7). Simple statistical measures, such as N50 values and the mean contig length, were smaller than those in many studies involving transcriptome assemblies, mainly because we analysed contigs with a minimum length of 100 bps instead of 200 bps (the default cut-off in the Trinity assembler). Several genes of interest (for example, the plastid genes *rpl32* and *rpl36*) are shorter than 200 bps, and use of the default cut-off would incur a risk of missing their transcripts. The assembly statistics were also biased by contamination. The symbiosis of mycoheterotrophic plants with fungi may be quite deep; for example, the rhizome of *E. aphyllum* has been described as “heavily and permanently infected” [46]. Despite the fact that we sequenced RNA from the above-ground parts of the plants, we observed a large number of bacterial and fungal transcripts in the assemblies (Additional file 5: Figure S1), especially in *E. roseum*, in which they represented ~ 80% of contigs that we were able to taxonomically classify by BLAST. In *E. aphyllum* and *H. monotropa*, the corresponding values were approximately 10 and 5%, respectively. This difference may reflect the correlation between soil microbiome biomass and climate (*E. aphyllum* and *H. monotropa* were collected from colder regions than *E. roseum*). Among the transcriptomes of photosynthetic plants used for comparison, the transcriptome of *O. italica* was also highly contaminated. The high amount of contamination reduces the number of reads belonging to true plant transcripts in *E. roseum* and *O. italica*. Nevertheless, we do not consider this a significant obstacle since *E. aphyllum* (which does not have a lot of contamination) and *E. roseum* are very close phylogenetically and similar gene content is expected in their genomes. Likewise, using *P. equestris* and *C. ensifolium* as two additional reference photosynthetic orchids compensates for a potentially poor assembly of the *O. italica*. Sequences originating from fungi and bacteria were discarded prior to further analysis. Estimation of the completeness of the assemblies based on the set of genes that are expected to be present in all eukaryotic genomes [27] showed that > 90% of these genes were at least partially assembled (Additional file 1: Table S8). Notably, the genomes were assembled less completely than the transcriptomes on average, with a median of 95.6% of the genes being assembled at least partially in the transcriptome assemblies, compared with 90.8% of the genes in the genome assemblies. These results show that, given sufficient coverage, RNA-seq is as good as complete genome sequencing in terms of the number of retrieved genes, while being less costly and using an assembly process that is computationally faster. It could however miss some genes expressed at very low levels, thus the results on gene loss based on transcriptome assemblies should be interpreted with more caution.

**Table 1:** Brief statistics of the transcriptome assemblies

	*Epipogium aphyllum*		*Epipogium roseum*		*Hypopitys monotropa*	
	All transcripts	CDSs^a^	All transcripts	CDSs^a^	All transcripts	CDSs^a^
Number of sequences	992,338	20,958	1,336,170	19,026	217,166	13,276
Number of sequences longer or equal to 1000 bps	39,403	4321	28,284	3259	33,560	5421
Total length of sequences	290,947,719	12,988,731	321,048,790	10,721,418	116,614,033	13,496,709
N50	371	1173	257	990	1199	1470
Median length of sequences	178	306	164	315	237	807

^a^Here, the CDS is defined as the longest ORF in the major isoforms of the transcripts that were assigned at least one GO term (low-coverage transcripts and contaminating transcripts are not considered)

### Gene retention and reduction

Plastid-targeted proteins carry specific amino acid sequences known as targeting signals or transit peptides that interact with the translocon system and enable the import of these proteins into plastids. Thus, we expected that in non-photosynthetic plants, the fraction of proteins with plastid-targeting signals relative to other proteins will be decreased. However, a comparison revealed a more complex situation. In *Epipogium*, the fraction of proteins targeted to plastids relative to the total number of proteins is approximately 2 times lower than in autotrophic orchids on average, whereas the fraction of proteins that are targeted to mitochondria or the endoplasmic reticulum is approximately the same. In contrast, in *H. monotropa*, the fraction of proteins targeted to plastids does not appear to be decreased. However, because the fraction of plastid-targeted proteins differs greatly between the two photosynthetic Ericales species that we used for comparison (Additional file 1: Table S9), these results should be treated with caution, as they may be biased by lineage-specific genome duplications and/or by differences in the quality of the assemblies. To obtain a deeper understanding of the patterns of gene reduction, we performed Gene Ontology (GO) enrichment analyses. GO analysis of *Epipogium aphyllum* and *E. roseum* versus three photosynthetic orchids revealed that the genes associated with 60 GO terms in *Epipogium* were underrepresented, while the genes associated with 38 terms were overrepresented, with q-values ≤0.05. All of the overrepresented GO terms are related to genes associated with mobile elements. Although the titles of these GO terms (like “RNA-dependent DNA replication” or “endoribonuclease activity”) do not directly mention mobile elements, we checked several dozen random genes with these GO terms and verified by BLASTP alignment to the NCBI NR database that many of them are indeed genes of mobile elements. Some high-level (in terms of the Gene Ontology hierarchy) terms are overrepresented probably because they include low-level terms describing genes of mobile elements, for example the term “nucleobase-containing compound metabolic process” includes the term “RNA-dependent DNA replication”. The presence of overrepresented GO terms related to mobile elements is presumably caused by methodological differences, as the *Epipogium* transcriptomes were sequenced without selection of polyadenylated transcripts, whereas the transcriptomes of *Cymbidium ensifolium* and *O. italica* represented polyA fractions. Thus, mobile elements whose RNAs are rarely polyadenylated (for example, like the mobile elements of the BARE family [47]), are expected to be overrepresented in *Epipogium*. In the genome of *Phalaenopsis equestris*, mobile elements are masked as repeats, producing a similar effect. Among the underrepresented GO categories, almost all were related to photosynthesis and plastids. The least underrepresented GO terms were general and difficult to interpret (e.g., “Single-organism metabolic process” and “Membrane”). The most underrepresented GO terms are listed in Table 2; for a complete list, see Additional file 1: Table S6.

**Table 2:** GO terms showing the greatest differences in the fraction of genes between mycoheterotrophic species and their photosynthetic relatives

*Epipogium* compared with photosynthetic orchids				
GO term	Type of GO term	Median number of genes with this GO term in *Epipogium aphyllum* and *Epipogium roseum*	Median number of genes with this GO term in photosynthetic orchids	Median ratio of fractions between *Epipogium aphyllum* and *Epipogium roseum* versus photosynthetic orchids^a^
chlorophyll binding	molecular function	0.5	34	0.011
photosynthesis, light harvesting	biological process	1	23	0.035
photosystem I	cellular compartment	2	38	0.041
plastid thylakoid lumen	cellular compartment	1.5	31	0.041
plastid thylakoid membrane	cellular compartment	55.5	201	0.20
photosynthesis, light reaction	biological process	50	174	0.22
plastid thylakoid	cellular compartment	89	275	0.25
photosystem II assembly	biological process	28	93	0.25
photosynthesis	biological process	125	294	0.35
organelle subcompartment	cellular compartment	203.5	403	0.38
*Hypopitys monotropa* compared with photosynthetic Ericales				
GO term	Type of GO term	Number of genes with this GO term in *H. monotropa*	Median number of genes with this GO term in photosynthetic Ericales	Median ratio of fractions between *H. monotropa* versus photosynthetic Ericales^a^
plastid thylakoid membrane	cellular compartment	51	247	0.34
photosynthesis, light reaction	biological process	51	243	0.37
plastid thylakoid	cellular compartment	80	341	0.39
photosynthesis	biological process	97	347	0.47
tetrapyrrole binding	molecular function	136	395	0.57
plastid	cellular compartment	1051	2177	0.81
oxidation-reduction process	biological process	1122	2256	0.83
single-organism process	biological process	6479	11,495	0.94

GO terms that are similar to each other, such as “plastid thylakoid lumen” and “chloroplast thylakoid lumen”, are combined here

^a^The fraction of genes with a specific GO term is the number of genes with that GO term in a species divided by the total number of genes with GO terms in that species. The median ratio of fractions is a measure of the difference in the numbers of genes with a specific GO term between two groups of species, calculated as a median value among all pairwise comparisons in which the first member in a pair comes from the first group, and the second member of the pair comes from the second group

GO enrichment analyses between *H. monotropa* and three photosynthetic Ericales showed similar results; 17 GO terms were overrepresented, and nine are underrepresented. The overrepresented terms were related to mobile elements, and the underrepresented terms were related to photosynthesis and plastids (Table 2; Additional file 1: Table S6).

Notably, we did not observe changes in the list of GO terms other than related to photosynthesis and plastids, despite dramatic differences in the morphology and physiology of the studied species. This finding indicates that these differences are controlled not at the level of the presence or absence of specific genes, but rather by the regulation of gene expression. To gain insight into this regulation, more detailed transcriptome data are required. Alternatively, these morphological and physiological changes may have originated from the disruption or loss of a small number of genes which do not produce statistically significant results in GO enrichment analysis.

The statistical analysis of GO terms is quite rough method and may not reflect differences at the level of individual genes. Thus, we also searched for orthologs of genes that are known to participate in processes occurring in the plastids in the model plant *Arabidopsis thaliana*.

As expected, genes related to photosynthesis have been lost from nuclear genomes of *Epipogium* and *H. monotropa* (Table 3). In particular, no nuclear-encoded components of the plastid electron transfer chain (*PSB* and *PSA* genes) were found, which is consistent with the absence of plastid-encoded *psa* and *psb* genes from the plastomes of all three species. Components of the light-harvesting antenna (*LHC* genes) were completely absent from *H. monotropa*; one such gene (*LHCA4*) was present in the *E. aphyllum* transcriptome, but its expression (measured in FPKM) was ~ 7–25-fold lower than in photosynthetic orchids. Nevertheless, this gene exhibited a full-length open reading frame that has evolved under negative selection (dN/dS 0.11). Since the product of this gene participates in chlorophyll binding, its retention may be in some way related to the retention of chlorophyll synthesis in *Epipogium*, as described below. Additionally, as shown in Additional file 1: Table S10, a few transcripts of genes that encode proteins in the plastid electron transfer chain were found in the transcriptomes of *Epipogium aphyllum*, *E. roseum* and *H. monotropa*. All of the observed sequences were shorter than 50% of the length of their orthologs in photosynthetic plants and are likely to be pseudogenes. The apparent partial conservation of enzymes in the Calvin cycle is due to that some of these enzymes are involved in metabolic processes that are not related to photosynthesis, e.g., glycolysis. Many genes that encode Calvin cycle enzymes belong to small gene families (e.g., *GAPDH*, *PGK*, *TKL*, *TPI*) in which different members encode proteins that exhibit the same enzymatic activity (isoenzymes) but show different cellular localizations and act in different pathways. For example, the GAPDH and TPI proteins function in both glycolysis (cytosolic isoenzymes) and the Calvin cycle (plastidic isoenzymes). We assume that most of the transcripts corresponded to cytosolic isoenzymes. Consistent with this assumption, 8 of 10 of the transcripts found in *Epipogium* and 7 of the 14 transcripts found in *H. monotropa* did not have plastid-targeting signals. Most of the transcripts that did not have plastid-targeted signals have the 5′-ends of their CDSs completely assembled (according to the TransDecoder predictions), so we don’t suppose this to be a result of misassembly. The transcripts with plastid-targeting signals (*TKL*, *RPI*) exhibit additional functions in plastids that are not related to the Calvin cycle. Expression of genes encoding proteins that act exclusively in photosynthesis (e.g., rbcS, SBPase) was absent. The nuclear-encoded components of plastid ATP synthase have been completely lost, as well as the plastid-encoded components. The sigma subunits of plastid-encoded RNA polymerase (PEP) and PEP-associated proteins have also been lost. PEP is involved in the transcription of genes related to photosynthesis, unlike nuclear-encoded RNA polymerase (NEP), which mainly transcribes plastid genes that are unrelated to photosynthesis [48]. The redox-sensitive components of the plastid translocon, which is the system responsible for the import of proteins from the cytoplasm to plastids, have also been lost, with the exception of TIC32 of *Hypopitys*. This was expected, since the redox-sensitive components of the plastid translocon are thought to be regulated by redox signals from photosynthesis [49]. Transcripts encoding some components of the plastid translocon (as well as some other proteins analysed in the Additional file 1: Table S10) which are present in *A. thaliana* are absent in all studied Orchidaceae and Ericales. This indicates that not all cases of gene absence in *Epipogium* and Hypopitys should be ascribed to the loss of photosynthesis.

**Table 3:** Number of selected photosynthesis-related nuclear genes and nuclear genes encoding proteins involved in plastid functions other than photosynthesis in *Epipogium aphyllum* and photosynthetic orchids as well as *Hypopitys monotropa* and photosynthetic Ericales

Genes	Number in *E. aphyllum*	Median number in photosynthetic orchids	Number in *H. monotropa*	Median number in photosynthetic Ericales
Photosynthesis-related				
Components of photosystem I	0	9	0	8
Components of photosystem II	0	9	0	8
Components of electron transfer chain (others than PSI and PSII)	0	5	0	6
Light-harvesting complex	1	10	0	8
Calvin cycle	10	20	14	21
Sigma subunits of PEP and PEP-associated proteins	0	14	1	14
Plastid ATP synthase	0	3	0	3
Non-photosynthesis-related				
Plastid ribosome	33	34	31	31
Clp subunits	11	12	10	10
ACC subunits	5	6	4	7
Plastid translocon	15	20	11	18

In contrast to genes encoding proteins that are necessary for photosynthesis, genes that are responsible for other functions of plastids have been retained (Table 3). In particular, components of clp-protease and acetyl-CoA carboxylase whose counterparts (clpP and accD) are encoded in the plastome have been retained. Consistent with this finding, NEP, which transcribes genes not related to photosynthesis, has been retained in both species, as have most components of the plastid ribosome (but see the discussion below). We also found transcripts of most proteins responsible for the replication and repair of the plastome. However, this situation is more complex because many of these proteins are targeted not only to plastids but also to mitochondria and the nucleus, and information on these proteins is sometimes inconsistent between different experiments [50, 51]. A protein responsible for recombination and repair which we observed in almost all photosynthetic species of comparison but did not observe in all heterotrophic species is RECA1. The search for its homologs suggests that there are no plastid-targeting homologs of RECA in *E. aphyllum* and *E. roseum*. There is one homolog in *H. monotropa* for which predictions of TargetP and DualPred are contradictory, with TargetP suggesting no transit peptide and DualPred suggesting plastid targeting. As RECA participates in plastid recombination and repair (reviewed in [52]) the loss of RECA may explain both the increase in the substitution rate of the plastid genomes and the increase in their AT-content [11, 18], as gene conversion in plastids is GC-biased [53–55].

As shown above, the genomes of *Epipogium* and *H. monotropa* encode a number of proteins that must be imported into plastids. Accordingly, the components of the plastid translocons for both the outer- and inner-envelope membranes must be retained. Recent studies in *A. thaliana* have shown that the plastid-encoded protein ycf1 (TIC214) plays an essential role in plastid translocation and that it acts at the inner membrane [56]. However, the *ycf1* gene is absent from both the *Epipogium* and *H. monotropa* plastomes. A transcript similar to *ycf1* was only found in the transcriptome assembly of *E. aphyllum*, in which it carried a signal for targeting of the protein to mitochondria. Homologs of TIC100 and TIC56, which encode proteins that interact with Ycf1 within the TIC complex, were also absent from all three species. It should be noted that Ycf1 and its interacting proteins are also absent from several photosynthetic species, including grasses and *Vaccinium macrocarpon*, which raises a question regarding the universality of the function of Ycf1 [57]. The current model of TIC postulates the existence of two complexes. The first, referred to as the “photosynthetic-type” complex, consists of Ycf1, TIC56, TIC100 and TIC20-I and is a major TIC complex that functions in most land plants to import proteins involved in photosynthesis. The second complex is a “non-photosynthetic-type” complex, which imports proteins that are not related to photosynthesis [58]. It has been hypothesized that the switch from the major to the alternative system of protein import occurred in grasses [58, 59] and that the major system then degraded. The pattern of gene loss and retention observed in *Epipogium* and *H. monotropa* suggests that this could also be the case in these species. The existence of two complexes, one of which mainly imports photosynthetic proteins, while the other is non-photosynthetic, has also been postulated for the outer translocon TOC [58, 59]. *Epipogium* and *H. monotropa* possess orthologs of TOC proteins, but not a complete set (Table 2), suggesting that only one of these complexes is retained in these plants.

Annotation of the transcriptomes using the KEGG database of biological pathways revealed reductions in the number of genes associated with several other pathways, some of which are common to *Epipogium* and *H. monotropa*, while others are lineage-specific (Additional file 6: Figure S2, Additional file 7: Figure S3, Additional file 8: Figure S4 and Additional file 9: Figure S5). For example, a reduction in the number of proteins involved in light reception and circadian rhythms was observed; although this reduction was found in *Epipogium* as well as in *Hypopitys*, it was notably different in these fully heterotrophic plants (Additional file 6: Figure S2). In particular, *Epipogium* appears to lack genes encoding the photoreceptors phytochrome B and cryptochrome, whereas *H. monotropa* lacked several proteins (LHY, ZTL and GI) that regulate the circadian clock. The absence of these proteins may be related to the distinctive lifestyles of these plants, which spend most of their life cycle completely underground and appear above-ground only for several weeks during blossoming [60–62], and may therefore have different requirements for the regulation of circadian rhythms than typical autotrophic plants. However, both *Epipogium* and *H. monotropa* have retained essential elements of circadian clock regulation (HY5, ELF3), photoreceptor phytochrome A and proteins interacting with them (PIF, COP1), indicating that the core proteins that regulate plant development under the influence of light have been conserved. In addition, we found a reduction in the number of genes associated with the carotenoid (Additional file 7: Figure S3) biosynthesis pathway. As carotenoids are a component of light-harvesting antennae, the reduction in the number of genes related to carotenoid biosynthesis is presumably linked to the disappearance of photosynthesis. The pathway for phylloquinone synthesis is lost completely, while the pathway for plastoquinone synthesis persists (Additional file 8: Figure S4). Two functions of phylloquinone are known, namely it participates in the plastid electron-transfer chain and also aids in forming disulphide bond in PsbO [63]. As PsbO is a protein involved in photosynthesis, phylloquinone is dispensable in non-photosynthetic plants. Plastoquinone is a part of the plastid electron-transfer chain, but also it has other functions, for example it acts as an antioxidant [64], mediates stress response [65] and participates in synthesis of some plant hormones [66, 67]. This may explain the retention of the plastoquinone synthesis pathway.

**Fig. 2:**
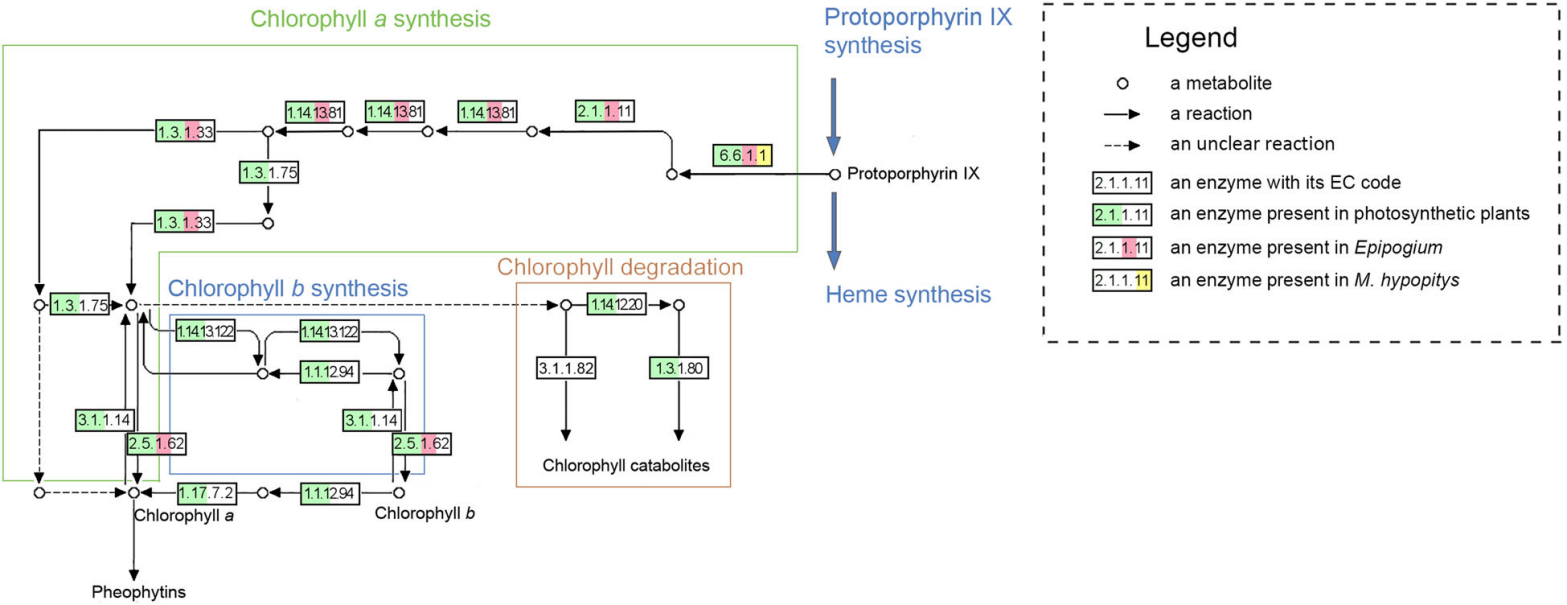
Map of the chlorophyll metabolism pathway showing proteins present in *Epipogium*, *Hypopitys monotropa* and green plants. A protein is depicted as present in a specific plant group if its transcript was observed in a transcriptome of at least one species of that group

Another contrasting characteristic was the retention of the chlorophyll *a* synthesis pathway in *Epipogium*, whereas only the gene responsible for the first step in this pathway has been retained in *H. monotropa* (Fig. 2). That gene (*ALB1*) codes for a protein which works in a complex with two other proteins (CHLI and GUN5) whose transcripts are not found in the transcriptome of *H. monotropa*. Thus, even the first reaction of the chlorophyll synthesis pathway is probably not accomplished in *H. monotropa*. The dN/dS of *ALB1* in *H. monotropa* is significantly higher than in autotrophic Ericales (*p*-value of 0.02 by the likelihood ratio test), showing a value of 0.27 in *H. monotropa* versus 0.11 in its autotrophic relatives. As the value in *H. monotropa* is lower than 1, negative selection on this gene may have disappeared only recently, or, alternatively, this gene may still have some unknown function. Among the 5 considered genes in *Epipogium*, only 1 showed increased dN/dS with a significant p-value (< 0.05 by the likelihood ratio test with Bonferroni correction). The mean dN/dS for these 5 genes in *Epipogium* was only slightly higher than the mean in its photosynthetic relatives, at 0.11 versus 0.05, respectively (Additional file 1: Table S10). However, all of the genes in this pathway were expressed at levels many times lower than in photosynthetic species (Additional file 1: Table S10). Chlorophyll *a* could theoretically be synthesized in *Epipogium*, where it may act in cellular processes that are unrelated to photosynthesis. Chlorophyll *a* has been found in many heterotrophic plants via chromatography [68] (notably, among these species is *Monotropa uniflora*, a close relative of *H. monotropa*); transcriptome sequencing in parasitic *Orobanche aegyptiaca* has also demonstrated the presence of genes responsible for chlorophyll *a* synthesis, with no signs of relaxed selection [17]. It has been shown that in *A. thaliana*, pheophorbide *a*, a product of chlorophyll *a* catabolism, causes cell death in a light-independent manner [69, 70]. Conservation of this mechanism in *Arabidopsis* and non-photosynthetic plants could explain the conservation of the chlorophyll *a* synthesis pathway.

**Fig. 3:**
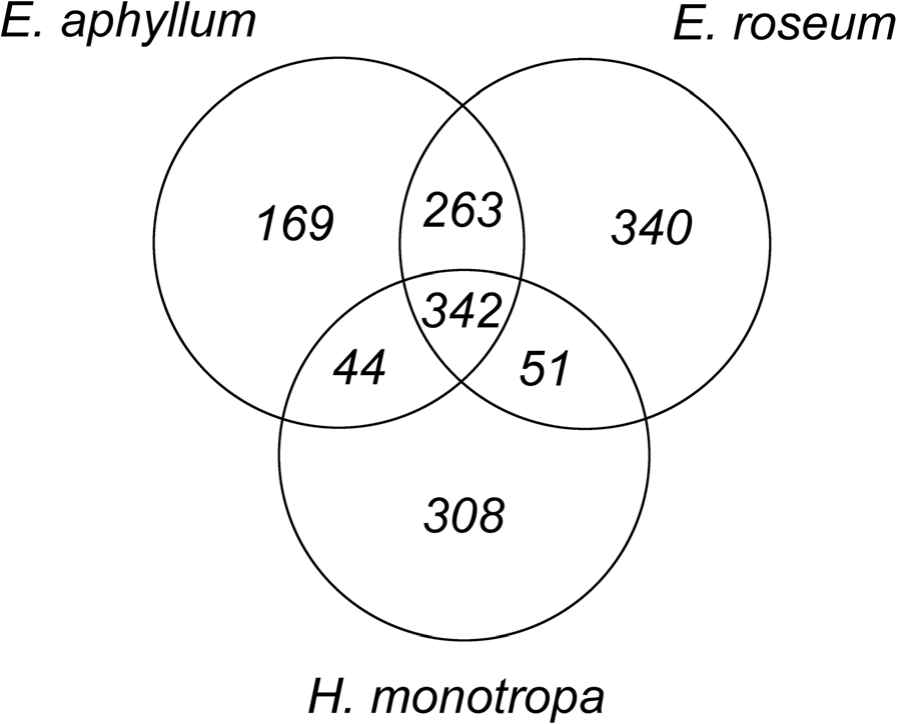
The Venn diagram representing numbers of genes absent from the transcriptome assemblies of the studied mycoheterotrophic species. Only the genes whose homologs were found in all 6 photosynthetic species of comparison are counted

Despite the differences discussed above, *Epipogium* and *H. monotropa* show striking parallelism in the reduction of their nuclear genes, considering that these plants lost autotrophic ability independently and that they diverged approximately 150 million years ago [71]. Quantitative estimates of this parallelism can be biased by differences in assembly quality and by lineage-specific gene losses that are not related to heterotrophy. To obtain an unbiased estimate, we considered only the genes that presented one-to-one orthologs in the *Arabidopsis* genome and were found in all 6 photosynthetic species used for comparison (4888 genes in total). Among these genes, 818 were absent in *E. aphyllum*, and 745 were absent in *H. monotropa*. In *E. roseum*, 996 genes were absent, which presumably reflects the lower quality of the assembly obtained for this species (Fig. 3). For *E. aphyllum* and *E. roseum*, the overlap between the lost genes was 67%, whereas for *E. aphyllum* and *H. monotropa*, it was 49%, and for *E. roseum* and *H. monotropa*, it was 45%. If the losses occurred randomly, we would expect a much lower (~ 16%) percentage of common losses. According to the hypergeometric test, the *p*-value for the null-hypothesis that losses are uncorrelated was < 10^− 100^ for both the *E. aphyllum*/*H. monotropa* and *E. roseum*/*H. monotropa* pairs.

### Substitution rates

**Fig. 4:**
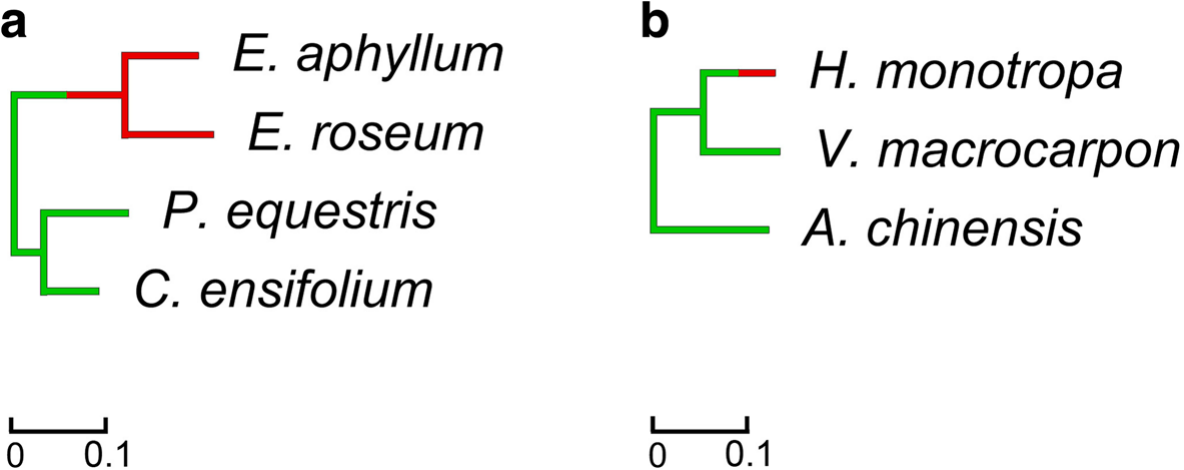
Substitution rates in nuclear genes of *Epipogium* (**a**) and *Hypopitys monotropa* (**b**). The branch lengths denote the number of nucleotide substitutions per position. Branches corresponding to non-photosynthetic species are indicated in red, and those corresponding to photosynthetic species are indicated in green. Branches in which a transition from a photosynthetic to a non-photosynthetic lifestyle occurred are indicated half in green and half in red. *Orchis italica* and *Camellia sinensis*, which are employed as outgroups in (**a**) and (**b**), respectively, are not shown, since the substitution rate of an outgroup cannot be evaluated

Variation in substitution rates is a phenomenon that is widely observed in plants (e.g., [72]. In particular, it has been found that in parasitic plants, the substitution rate is increased not only in plastome, as expected for heterotrophic plants, but also in nuclear and mitochondrial genomes [73]. Concerning mycoheterotrophic plants, information on substitution rates is mostly limited to plastomes, which show increased substitution rates with only rare exceptions [9, 74]. Our previous studies of *Epipogium* and *H. monotropa* indicated that the substitution rate in plastid genes, at both synonymous and in non-synonymous sites, was increased in comparison to the completely autotrophic relatives of these species, but at different levels (approximately 20 and 2.5 times respectively) [11, 18]. Characterization of transcriptome sequences allowed us to test whether this increase was confined to plastid genes. To calculate the substitution rate in the nuclear genomes, we used concatenated sequences of genes from orthogroups containing exactly one gene in each species. There were 4479 and 2547 of these orthogroups in Orchidaceae and Ericales, respectively. The substitution rates in both *Epipogium* species were approximately two times higher than the substitution rates in their photosynthetic relatives. The rates of non-synonymous and synonymous substitutions accumulation in *Epipogium* were increased proportionally (Additional file 10: Figure S6, Additional file 11: Figure S7 and Additional file 12: Figure S8). In contrast, the substitution rate in *H. monotropa* was not increased (Fig. 4).

The synonymous substitution rate (dS) estimate may be incorrect in cases where dS is large, due to saturation. However, the saturation starts to manifest itself on branches where dS is higher than 0.5 [75], thus the saturation is unlikely to spoil our estimates of dS and dN/dS.

PAML estimates may also be biased when analyzing sequences with different GC-content (confirmed in a personal communication with Ziheng Yang, the author of PAML). Nevertheless, the values of GC-content of the studied species differ by at most several percent (see the Additional file 1: Table S11) and we do not consider the difference as a source of a potential problem.

### Composition of the plastid ribosome

The proteins of the plastid ribosome are encoded in both the plastid and nuclear genomes. For example, in *Arabidopsis thaliana*, 21 plastid ribosomal proteins are encoded by the plastome, and 36 are encoded by the nuclear genome. In all fully heterotrophic plants with highly reduced plastomes, some ribosomal genes are missing; *Pilostyles aethiopica*, in which only two ribosomal protein genes are retained, represents the most extreme known case [8]. This raises the question of how ribosomes are able to function in these species. There are three possibilities. First, some proteins of the plastid ribosome may simply be non-essential, and their loss may not severely affect ribosome function [76], although this does not explain such extreme cases of reduction. Second, since the transfer and integration of plastid DNA into the nucleus exists in plant cells, functional copies of plastid genes can arise in the nuclear genome. There are examples in which the products of such nuclear copies are targeted to plastids and function as a part of the plastid ribosome, while the corresponding gene having been lost from the plastome [77–79]. Third, components of mitochondrial ribosomes can be dually targeted to both plastids and mitochondria [80, 81]. In *E. aphyllum* [11], 7 ribosomal protein genes (*rpl20*, *rpl22*, *rpl23*, *rpl32*, *rpl33*, *rps15*, *rps16*) have been lost from the plastome; in *E. roseum* 6 of these 7 genes, but not *rpl20*, have been lost. Regarding *H. monotropa*, we previously considered *rps19* and *rpl22* to be pseudogenes [18] due to the presence of a 111-bps insertion in the former and a nonsense mutation that shortens the length of the product by 17% in the latter. However, because these genes are transcribed and exhibit dN/dS values close to those of the species’ photosynthetic relatives (Additional file 1: Table S10), we assume that they are functional genes. Two genes, *rps15* and *rps16*, were completely absent from the plastome of *H. monotropa*. We do not observe any transcripts with high similarity to these plastid genes in the transcriptomes of *E. aphyllum*, *E. roseum* or *H. monotropa*. Thus, the loss of these genes from the plastome is unlikely to be compensated by transfer of plastid sequences to the nuclear genome.

Instead, we found that several transcripts that are not 1–1 orthologs of plastid-encoded ones but have more distant homology to them encode proteins that can be imported into plastids. This is the case for Rpl23 and Rps15 in *E. aphyllum* and Rps15 in *H. monotropa*. Additionally, for several proteins, the predictions made with TargetP and DualPred, two tools that we used for target prediction, were contradictory. Specifically, for a homolog of Rpl32 in *E. aphyllum* and homologs of Rpl23, Rps15 and Rps16 in *E. roseum*, plastid targeting was predicted by only one of the two tools. In all cases except for Rpl23 of *E. aphyllum*, analysis of the transit peptides of the homologs in the photosynthetic relatives of these species suggested that these proteins were already targeted to plastids prior to the divergence of non-photosynthetic and photosynthetic species, which may have facilitated the loss of the respective plastid genes. Some of the aforementioned proteins are predicted to be targeted solely to plastids, and some are predicted to be dually targeted to plastids and mitochondria (for details see Additional file 1: Table S12).

To determine whether the genes whose products may serve to replace the lost ribosomal proteins are encoded in mitochondrial or nuclear genomes, we used TBLASTN to align the proteins against contigs produced during the assembly of the plastomes of *E. aphyllum* and *H. monotropa* in our earlier studies [11, 18] respectively). We did not observe the sequences of those genes in the mitochondrial contigs and therefore conclude that they are located in the nuclear genomes.

## Conclusions

In this study, we analysed and compared the transcriptomes of the mycoheterotrophic plants *Epipogium aphyllum, E. roseum* and *Hypopitys monotropa*. Despite the fact that *Epipogium* and *H. monotropa* are very distantly related, belonging to the monocots and eudicots respectively, and that these species lost photosynthesis independently, we observed a remarkable level of parallelism involving the reduction and retention of similar functional groups of genes. Among the observed differences were a more profound reduction in the chlorophyll *a* synthesis pathway in *H. monotropa* and an increased substitution rate in *Epipogium*. Overall, since there are several hundred fully heterotrophic species of flowering plants [1], with many cases of independent transitions to fully heterotrophic lifestyle, it is necessary to sequence and analyse more fully heterotrophic species in addition to *Epipogium*, *H. monotropa* and *Orobanche aegyptiaca* to predict whether this parallelism is universal. Significant help in this determination may be provided by the “1000 Plants Project” [82], in which the sequencing of many fully heterotrophic and mixotrophic species is being performed. The question that remains unanswered in this study is the mode of gene loss – did it occur through deletions of large regions carrying photosynthesis-related genes, or through the accumulation of mutations in the protein-coding and regulatory elements of these genes? We expect that characterization of the nuclear genomes of non-photosynthetic plants will fill this gap.

## Additional files

Additional file 1: **Table S1.** Information on transcriptome data. **Table S2.** Sources of genome sequences. **Table S3.** List of 1–1 orthologs between *Arabidopsis thaliana* and *Epipogium aphyllum*. **Table S4.** List of 1–1 orthologs between *Arabidopsis thaliana* and *Epipogium roseum*. **Table S5.** List of 1–1 orthologs between *Arabidopsis thaliana* and *Hypopitys monotropa*. **Table S6**. Complete list of GO terms for which the fractions of genes differed significantly among *Epipogium*, *Hypopitys monotropa* and their photosynthetic relatives. **Table S7.** Detailed statistics of the transcriptome assemblies. **Table S8.** Analysis of the completeness of the assemblies. **Table S9.** Proportions of genes whose products are targeted to various organelles relative to the total numbers of genes in the species according to TargetP analysis. Only genes with complete 5′ ends and at least one assigned GO term are considered. **Table S10.** Presence of genes of interest in the studied species and selective pressures acting on them. **Table S11.** GC-content of concatenated genes’ sequences. **Table S12.** Results of a search for possible substitutes for ribosomal proteins whose genes have been lost from the plastomes of *Epipogium* and *Hypopitys monotropa*. (XLSX 5745 kb)

Additional file 2: Longest open reading frames from major isoforms of the transcripts of *Epipogium aphyllum*, after removal of low-coverage transcripts and contamination. FASTA-headers were created by TransDecoder. (ZIP 7027 kb)

Additional file 3: Longest open reading frames from major isoforms of the transcripts of *Epipogium roseum*, after removal of low-coverage transcripts and contamination. FASTA-headers were created by TransDecoder. (ZIP 5621 kb)

Additional file 4: Longest open reading frames from major isoforms of the transcripts of *Hypopitys monotropa*, after removal of low-coverage transcripts and contamination. FASTA-headers were created by TransDecoder. (ZIP 6215 kb)

Additional file 5: **Figure S1.** Statistics regarding contamination in the studied transcriptomes. A total of 10,000 random transcripts (prior to the removal of low-coverage transcripts and searching for ORFs, but after the removal of minor isoforms) were taken from each assembly, and BLASTX alignment to NCBI NR (maximum allowed e-value of 10–5, word size of 3 amino acids, low-complexity sequence filter switched off) was performed. The transcripts were classified according to their best matches. In the distribution plots, the black lines denote median values, and the boxes denote interquartile ranges. (TIFF 1124 kb)

Additional file 6: **Figure S2.** Diagram of circadian rhythms regulation. (TIFF 967 kb)

Additional file 7: **Figure S3.** Diagram of carotenoid biosynthesis. (TIFF 563 kb)

Additional file 8: **Figure S4.** Diagram of ubiquinone and other terpenoid-quinone biosynthesis. (TIFF 422 kb)

Additional file 9: **Figure S5.** Diagram of thiamine metabolism. (TIFF 234 kb)

Additional file 10: **Figure S6.** Trees of the studied species with branch lengths representing dS (rate of synonymous substitutions). (TIFF 134 kb)

Additional file 11: **Figure S7.** Trees of the studied species with branch lengths representing dN (rate of non-synonymous substitutions). (TIFF 274 kb)

Additional file 12: **Figure S8.** Trees of the studied species with branch lengths representing dN/dS. (TIFF 330 kb)

## Abbreviations

bps: Base pairs
CDS: Coding sequence
FPKM: Fragments per kilobase per million reads mapped
GO: Gene ontology
HGT: Horizontal gene transfer
HMM: Hidden Markov model
kbps: Kilobase pairs
KEGG: Kyoto Encyclopedia of Genes and Genomes
NCBI: National Center for Biotechnology Information
ORF: Open reading frame
TAIR: The Arabidopsis Information Resource
SA: Transcriptome Shotgun Assembly Database
