# Supplementary figures and images for: RNA-seq highlights parallel and contrasting patterns in the evolution of the nuclear genome of fully mycoheterotrophic plants
Source: BMC Genomics. 2018 Aug 9;19:602. doi: 10.1186/s12864-018-4968-3 (PMC6085651; doi:10.1186/s12864-018-4968-3)

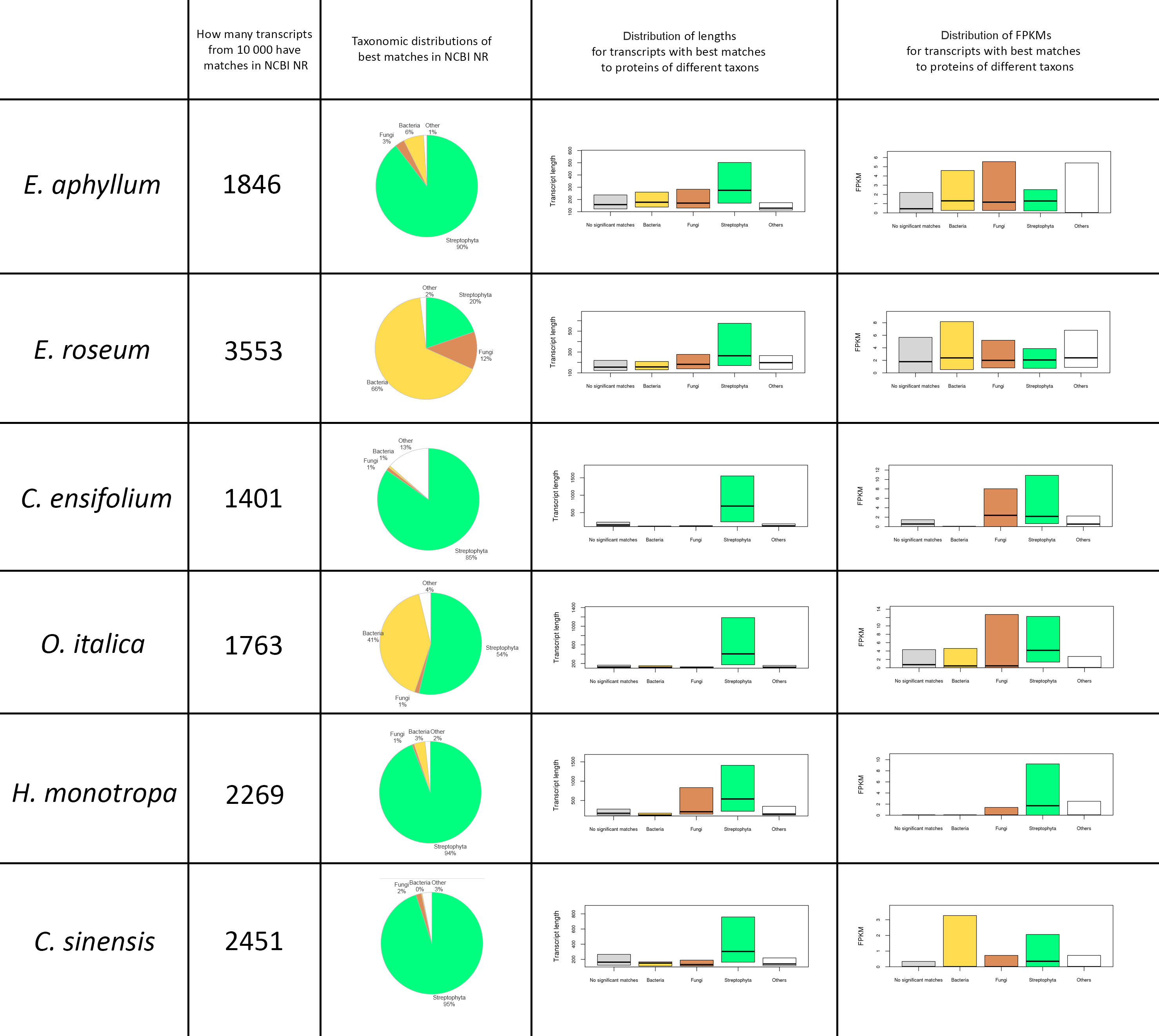

Supplement: Supplementary file 5 — Figure S1. Statistics regarding contamination in the studied transcriptomes. A total of 10,000 random transcripts (prior to the removal of low-coverage transcripts and searching for ORFs, but after the removal of minor isoforms) were taken from each assembly, and BLASTX alignment to NCBI NR (maximum allowed e-value of 10–5, word size of 3 amino acids, low-complexity sequence filter switched off) was performed. The transcripts were classified according to their best matches. In the distribution plots, the black lines denote median values, and the boxes denote interquartile ranges. (TIFF 1124 kb) [file 12864_2018_4968_MOESM5_ESM.tiff]

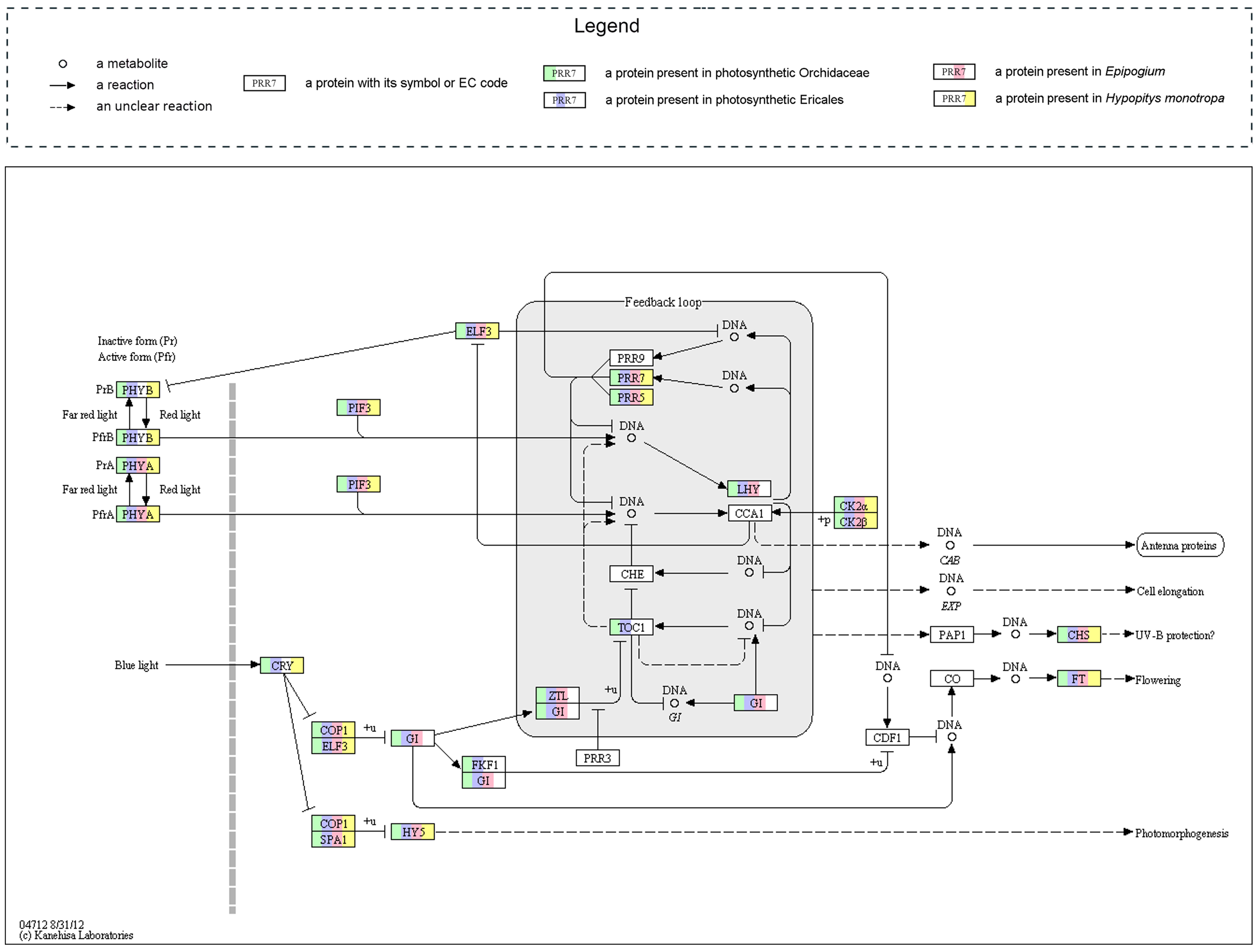

Supplement: Supplementary file 6 — Figure S2. Diagram of circadian rhythms regulation. (TIFF 967 kb) [file 12864_2018_4968_MOESM6_ESM.tiff]

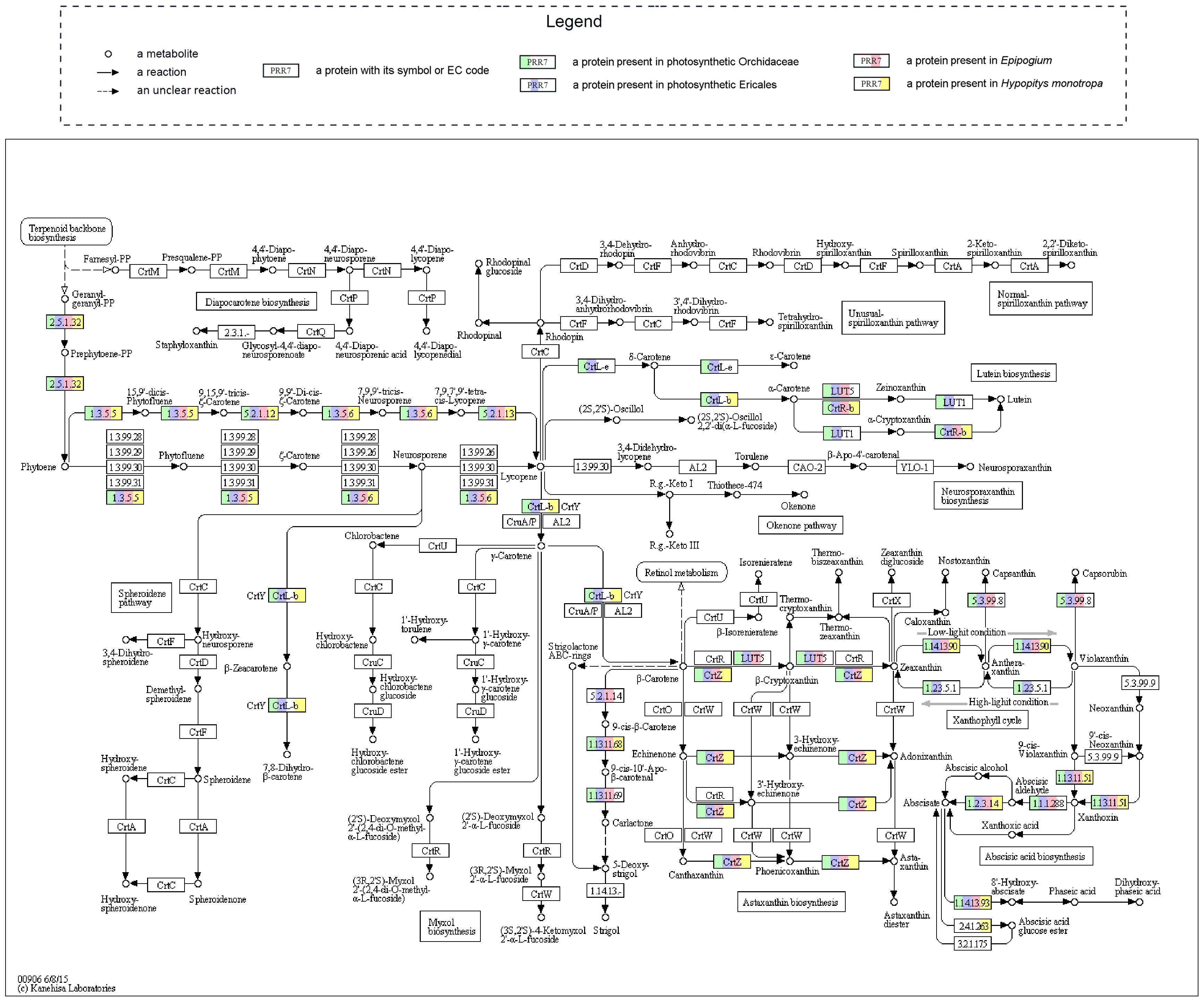

Supplement: Supplementary file 7 — Figure S3. Diagram of carotenoid biosynthesis. (TIFF 563 kb) [file 12864_2018_4968_MOESM7_ESM.tiff]

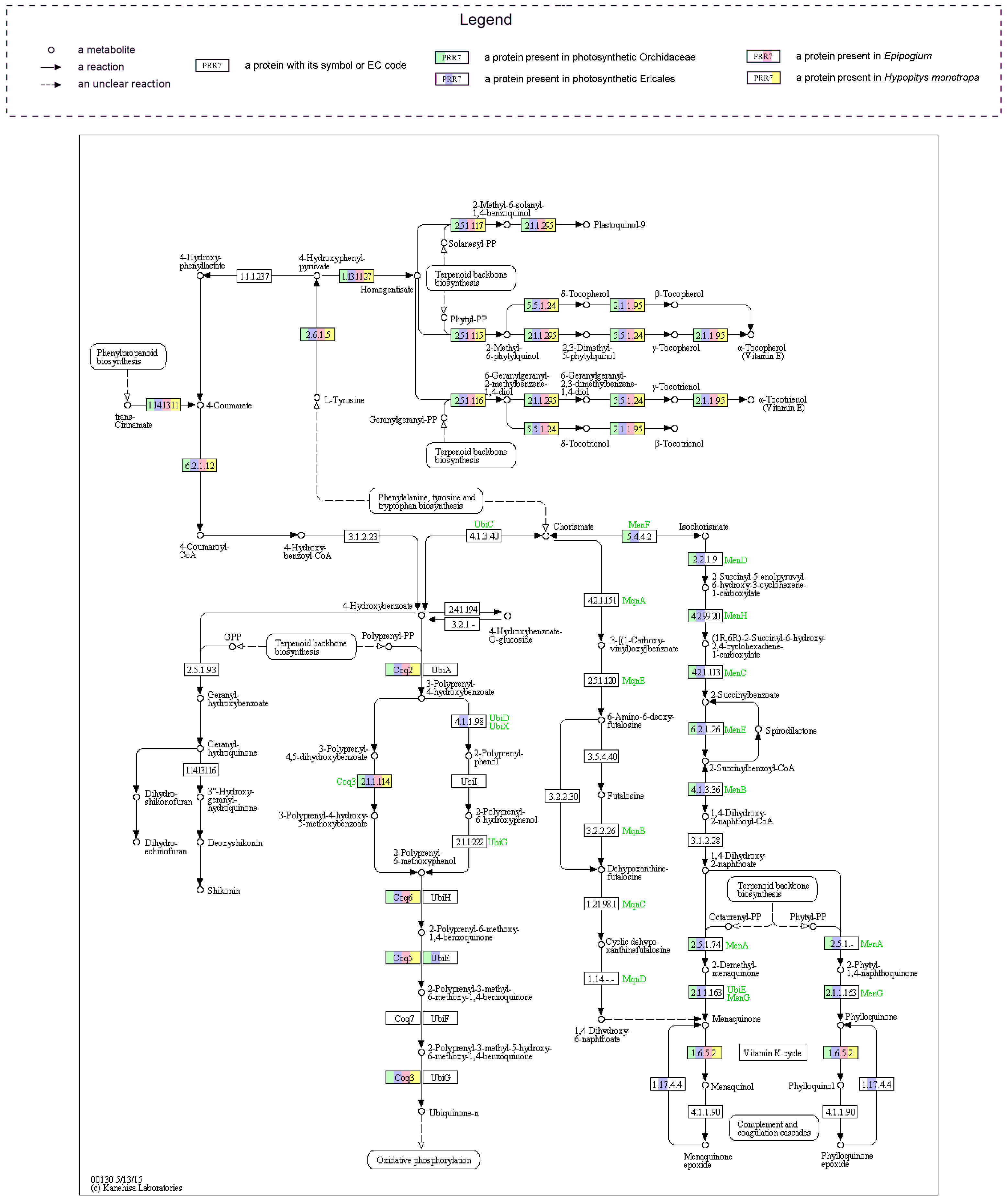

Supplement: Supplementary file 8 — Figure S4. Diagram of ubiquinone and other terpenoid-quinone biosynthesis. (TIFF 422 kb) [file 12864_2018_4968_MOESM8_ESM.tiff]

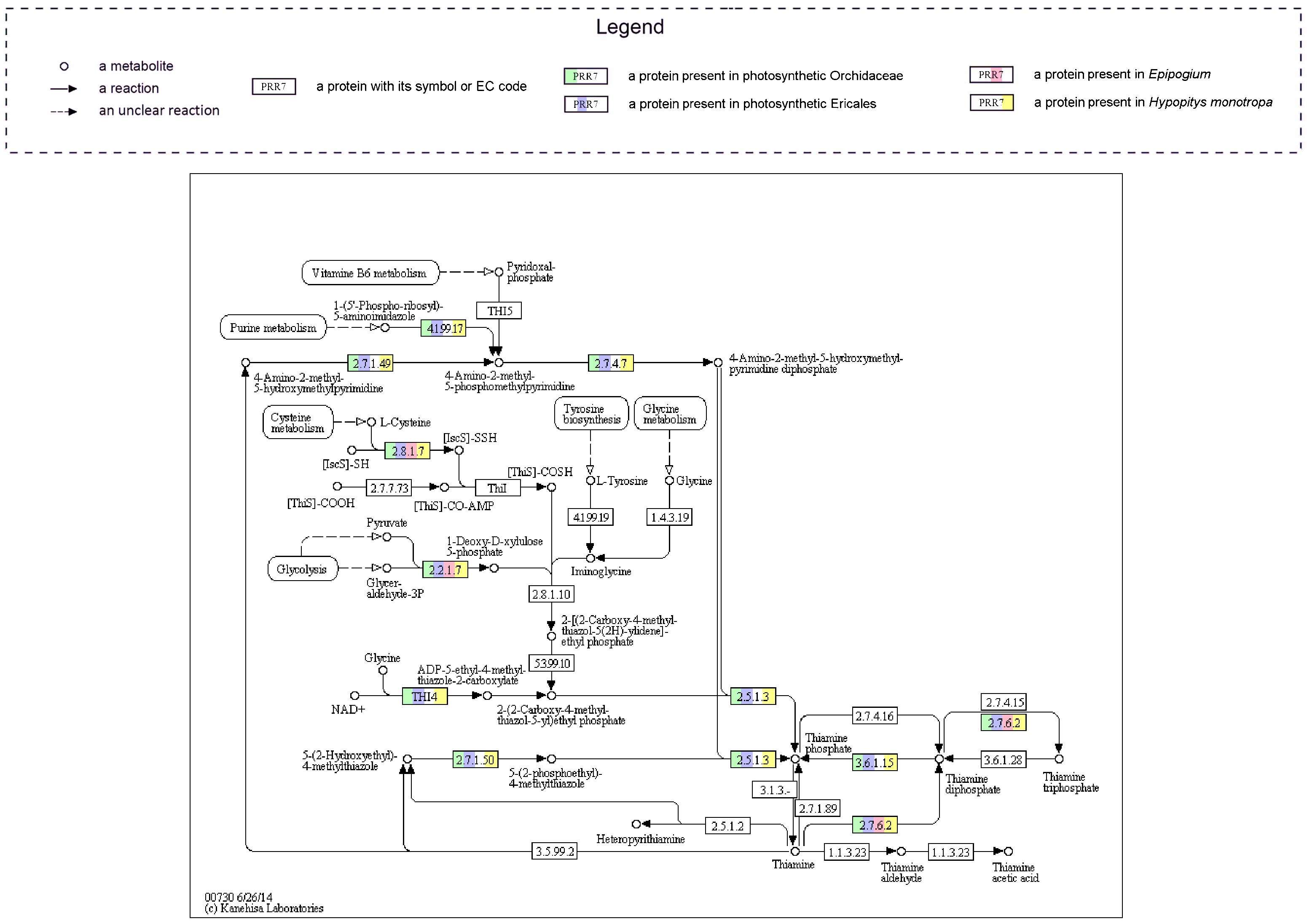

Supplement: Supplementary file 9 — Figure S5. Diagram of thiamine metabolism. (TIFF 234 kb) [file 12864_2018_4968_MOESM9_ESM.tiff]

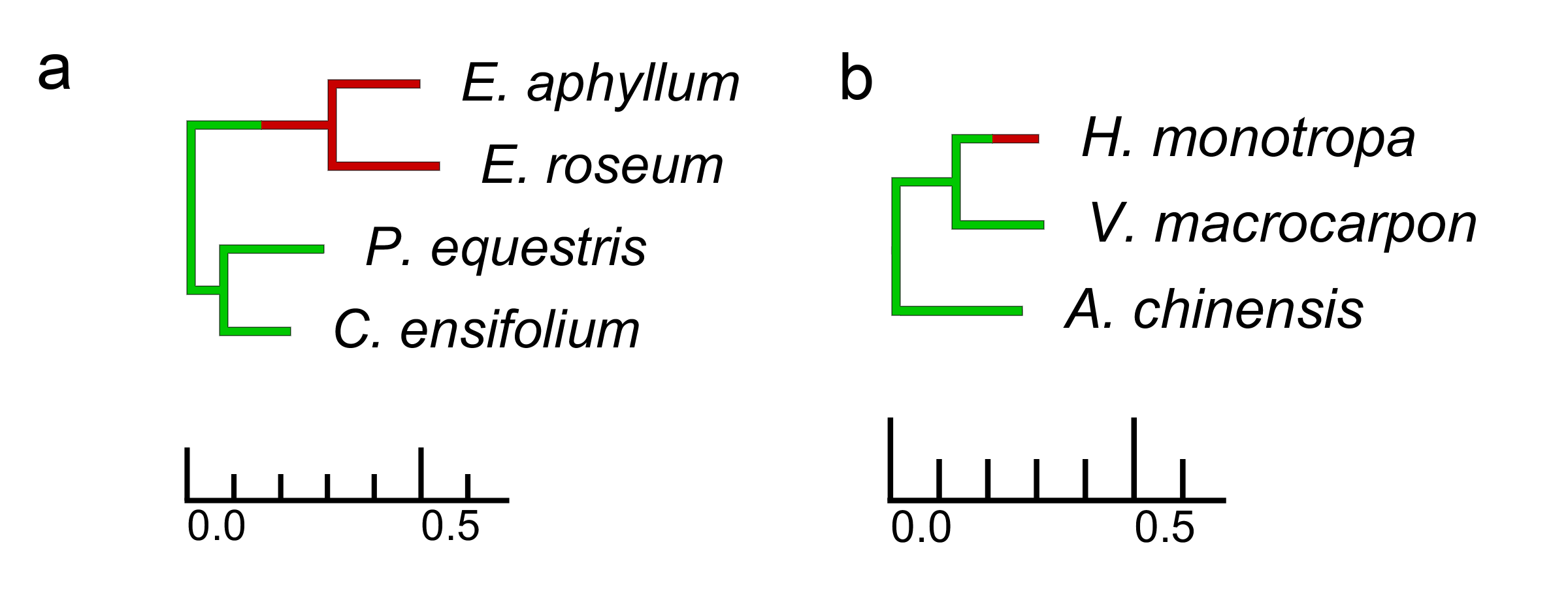

Supplement: Supplementary file 10 — Figure S6. Trees of the studied species with branch lengths representing dS (rate of synonymous substitutions). (TIFF 134 kb) [file 12864_2018_4968_MOESM10_ESM.tiff]

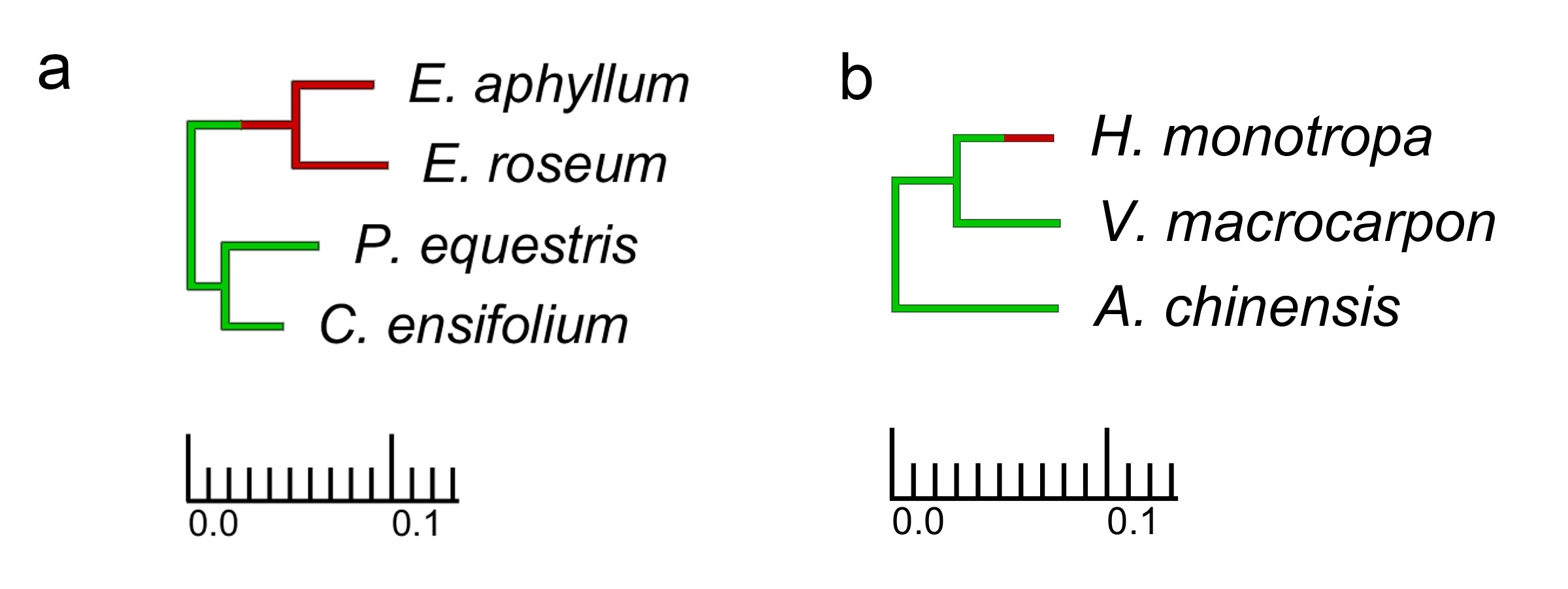

Supplement: Supplementary file 11 — Figure S7. Trees of the studied species with branch lengths representing dN (rate of non-synonymous substitutions). (TIFF 274 kb) [file 12864_2018_4968_MOESM11_ESM.tiff]

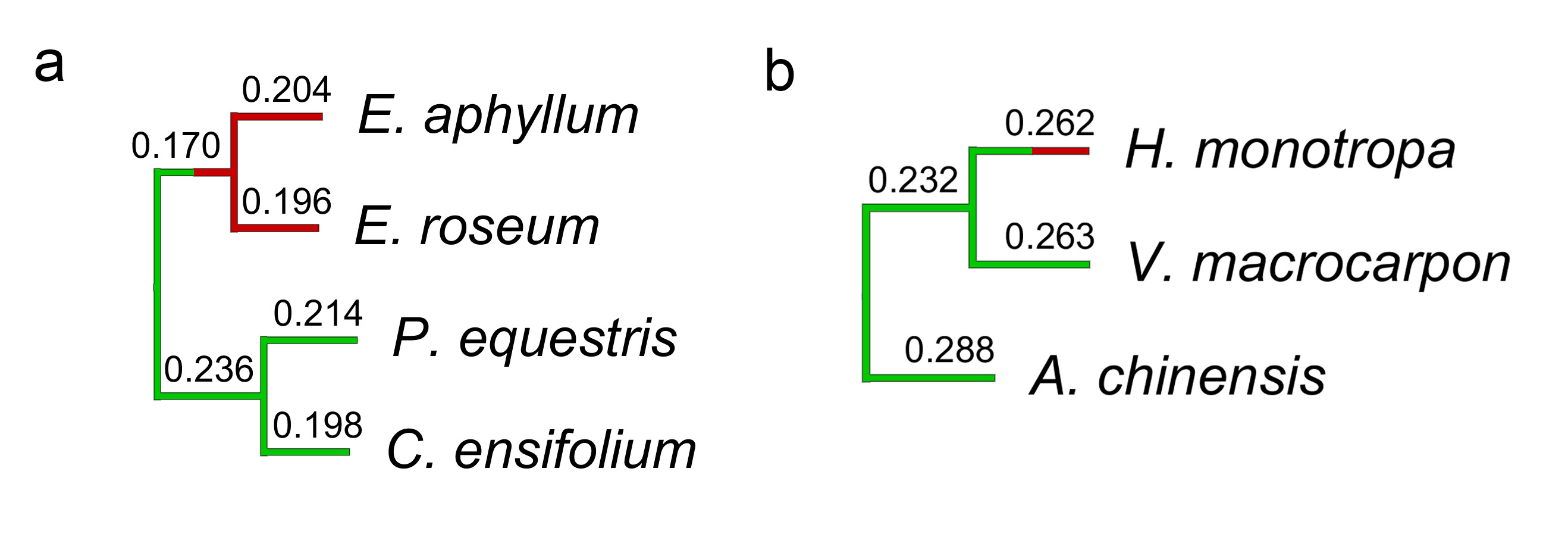

Supplement: Supplementary file 12 — Figure S8. Trees of the studied species with branch lengths representing dN/dS. (TIFF 330 kb) [file 12864_2018_4968_MOESM12_ESM.tiff]
